# The effect of the comorbidity burden on vitamin D levels in geriatric hip fracture

**DOI:** 10.1186/s12891-020-03554-1

**Published:** 2020-08-08

**Authors:** Ing How Moo, Carmen Jia Wen Kam, Eric Wei Liang Cher, Bryan Ce Jie Peh, Chung Ean Lo, David Thai Chong Chua, Ngai Nung Lo, Tet Sen Howe, Joyce Suang Bee Koh

**Affiliations:** 1 Department of Orthopedic Surgery, Singapore General Hospital, Singapore, Singapore; 2 Changi General Hospital, Singapore, Singapore

**Keywords:** Vitamin D, Charlson comorbidity index, Hip fracture

## Abstract

**Background:**

Elderly patients with hip fractures often have multiple medical comorbidities, and vitamin D deficiency is common in this population. Accumulating evidence links low vitamin D levels to various comorbidities. However, very little is known about the collective impact of comorbidities on vitamin D levels. The Charlson Comorbidity Index (CCI) is a validated comorbidity burden index. We hypothesized that a high CCI score is associated with vitamin D deficiency in elderly patients with hip fracture.

**Methods:**

A retrospective cohort study was conducted among all hospitalized elderly patients aged > 60 years admitted for low-energy hip fracture in a single tertiary hospital from 2013 to 2015. Data regarding patient demographics, fracture type, serum 25-hydroxyvitamin D3 levels and age-adjusted CCI score were collected and analysed.

**Results:**

Of the 796 patients included in the study, 70.6% (*n* = 562) of the patients were women and the mean age was 77.7 ± 8.0 years. The mean vitamin D level was 20.4 ± 7.4 ng/mL, and 91.7% ofhospitalized elderly patients with hip fracture had inadequate vitamin D level. There was no correlation between the individual serum vitamin D level with respect to age-adjusted CCI (Pearson correlation coefficient = 0.01; *p* = 0.87). After stratifying the CCI scores into low and high comorbidity burden groups (i.e., with scores 1–2 and ≥ 3), there was no relationship between the 2 subgroups for age-adjusted CCI and vitamin D levels (*p* = 0.497). Furthermore, there was also no association among age, gender, fracture type, and smoking status with the mean 25(OH)D level (*p* > 0.05).

**Conclusion:**

Low vitamin D levels were highly prevalent in our hip fracture cohort. There was no relationship between the CCI score and vitamin D levels in the geriatric hip population. The comorbidity burden in geriatric patients with hip fractures did not seem to be a significant factor for vitamin D levels.

## Background

Hip fracture often results in substantial morbidity, loss of independence, and mortality among the elderly population. Elderly patients with hip fractures often have multiple medical comorbidities, and only 4.9% of the elderly population with hip fracture patients reported no comorbidity [1]. Vitamin D deficiency is also highly prevalent among this population, ranging from 55 to 96.7% [2–4]. Even in the tropical country of Singapore, vitamin D deficiency was previously reported to be highly prevalent among hip fracture cohorts [5]. The high prevalence of vitamin D deficiency in elderly patients with multiple comorbidities that had hip fractures might not be just a coincidence.

Vitamin D deficiency contributes to increased fracture risk in the elderly population [4, 6, 7]. Suboptimal vitamin D levels are associated with impaired muscle strength and increased fall risks. It can also cause high bone turnover and low bone mineral density. In addition to its traditional effects on bone and mineral metabolism, accumulating evidence linking low vitamin D to various comorbidities involving nearly all organ systems as the vitamin D receptor has been found in many tissues outside the skeletal system [8–16].

Several investigations have reported a relationship between individual comorbidities and vitamin D deficiency [7, 9, 12–15, 17–21]. However, very little is known about the collective impact of comorbidities on vitamin D levels. The Charlson Comorbidity Index (CCI) is the most widely used method of assessing a patient’s overall comorbidity burden in clinical research [22]. The higher the score, the more severe the burden of comorbidity. Higher CCI scores have been shown to be associated with increased mortalities, length of stay and readmission in the geriatric hip fracture population [23–25].

To the best of our knowledge, no study has assessed the correlation of CCI with vitamin D deficiency. The purpose of this study was to use data from a large, level 1 trauma centre to explore the relationship between the CCI score and vitamin D deficiency. We hypothesized that a high CCI score is associated with low vitamin D levels in elderly patients with hip fractures.

## Materials and methods

This study (CIRB Ref: 2015/2134) was approved by the Singhealth Centralized Institutional Review Board, Singapore. We retrospectively studied data collected from January 2013 to December 2015 at a single tertiary hospital. The inclusion criteria were patients of aged > 60 years with femoral neck, intertrochanteric, or subtrochanteric fractures after a low-energy fall. The exclusion criteria were patients of age < 60 years, high-impact injuries, periprosthetic, peri-implant, pathological, and atypical femoral fractures. A retrospective review of the electronic medical records database was conducted for each patient. Data on patient demographics, including age, ethnicity, medical comorbidities, fracture type, CCI and serum 25-hydroxyvitamin D_3_ [25(OH)D] levels, were collected and analysed.

All patients were assessed using the age-adjusted CCI, calculated based on 17 comorbid conditions, with each assigned a weight of 1 to 6. The age-adjusted CCI takes into account each decade after 50 years of age as one point [26]. Based on the CCI score, the severity of comorbidity burden is usually classified into three grades: mild (CCI score 1–2), moderate (CCI score 3–4) and severe (CCI score ≥ 5). Accordingly, we considered the comorbidity burden as high grade if the CCI score was greater than or equal to 3 and low grade if the CCI score was less than 3.

The serum 25(OH)D levels were measured immediately upon admission using the radioimmunoassay method as part of the institution hip fracture protocol. In contrast to 1,25-dihydroxyitamin D, the serum 25(OH)D level is the best indicator of total vitamin D status, as it has a long half-life of 15 days and reflects the net incoming contributions from cutaneous synthesis and total dietary intake [27]. The serum vitamin D level was further categorized according to Holick’s classification, where vitamin deficiency is defined as a vitamin D level < 20 ng/mL, vitamin D insufficiency as a vitamin D level 21–29 ng/mL, and normal vitamin D levels are ≥30 ng/mL [7, 8].

### Statistical analysis

Statistical analysis was performed by a statistician using SPSS statistical software, version 19.0 (IBM Corp. Armonk, NY). Numerical data are presented as the mean and standard deviation, while categorical data are presented as frequency and percentage. The analysis sample was stratified into different groups by age, gender, smoking history, age-adjusted CCI, and individual comorbidities of the CCI. The mean levels of serum vitamin D were computed for each group. Comparisons within each group were performed using a 2-tailed *t*-test or one-way ANOVA. Pearson’s correlation coefficient was used to find the correlation between individual vitamin D levels and CCI. A two-tailed *p*-value of < 0.05 was considered statistically significant.

## Results

Of the 1087 patients with hip fracture admitted during the period from January 2013 to December 2015, 92% (*n* = 1002 patients) met the inclusion criteria. Data on vitamin D levels were available in 80% of the included patients (*n* = 796 patients) and were used in the analysis. Of the 796 patients included in the study, 562 (70.6%) were female. The mean age of the study population was 77.7 ± 8.0 years, and 83.6% of the patients were aged ≥70 years. The majority of the patients had femoral neck fractures (60.7%).

The mean vitamin D level of our study population was 20.4 ± 7.4 ng/mL. The prevalence of vitamin D deficiency among hospitalized elderly patients with hip fracture was 53.9% (*n* = 429), and vitamin D insufficiency was present in 37.8% of the patients (*n* = 301). Only 8.3% of all patients had normal vitamin D levels. Although there was no difference in vitamin D levels between Chinese and Malay patients, Indian patients had significantly lower vitamin D levels than the Chinese patients. Patient characteristics, demographic data, and distribution of 25(OH)D are summarized in Table 1.

**Table 1:** Patient characteristics, demographic data, and distribution of vitamin D levels

Variables	***n*** = 796	%	25(OH)D, ng/ml (mean ± SD)	***p***-value
Age (mean)				
60–69	131	16.5	20.0 ± 8.1	0.854
70–79	326	41.0	20.5 ± 6.8	
80–89	287	36.1	20.7 ± 7.6	
≥ 90	52	6.5	20.1 ± 8.0	
Gender				
Female	562	70.6	20.3 ± 7.5	0.615
Male	234	29.4	20.6 ± 7.0	
Race				
Chinese	702	88.2	20.7 ± 7.1*	0.012
Malay	54	6.8	18.8 ± 8.4	
Indian	32	4.0	16.6 ± 8.1*	
Others	8	1.0	22.0 ± 14.0	
Current smoker				
No	709	89.1	20.4 ± 7.5	0.884
Yes	87	10.9	20.3 ± 6.4	
Type of fracture				
Neck of femur	483	60.7	20.4 ± 7.3	0.485
Intertrochanteric	303	38.1	20.3 ± 7.5	
Subtrochancteric	10	1.3	23.2 ± 7.3	

*Bonferroni’s posthoc correction showed that the significant difference of Vitamin D level was between Chinese and Indian (*p* = 0.012)

**Fig. 1:**
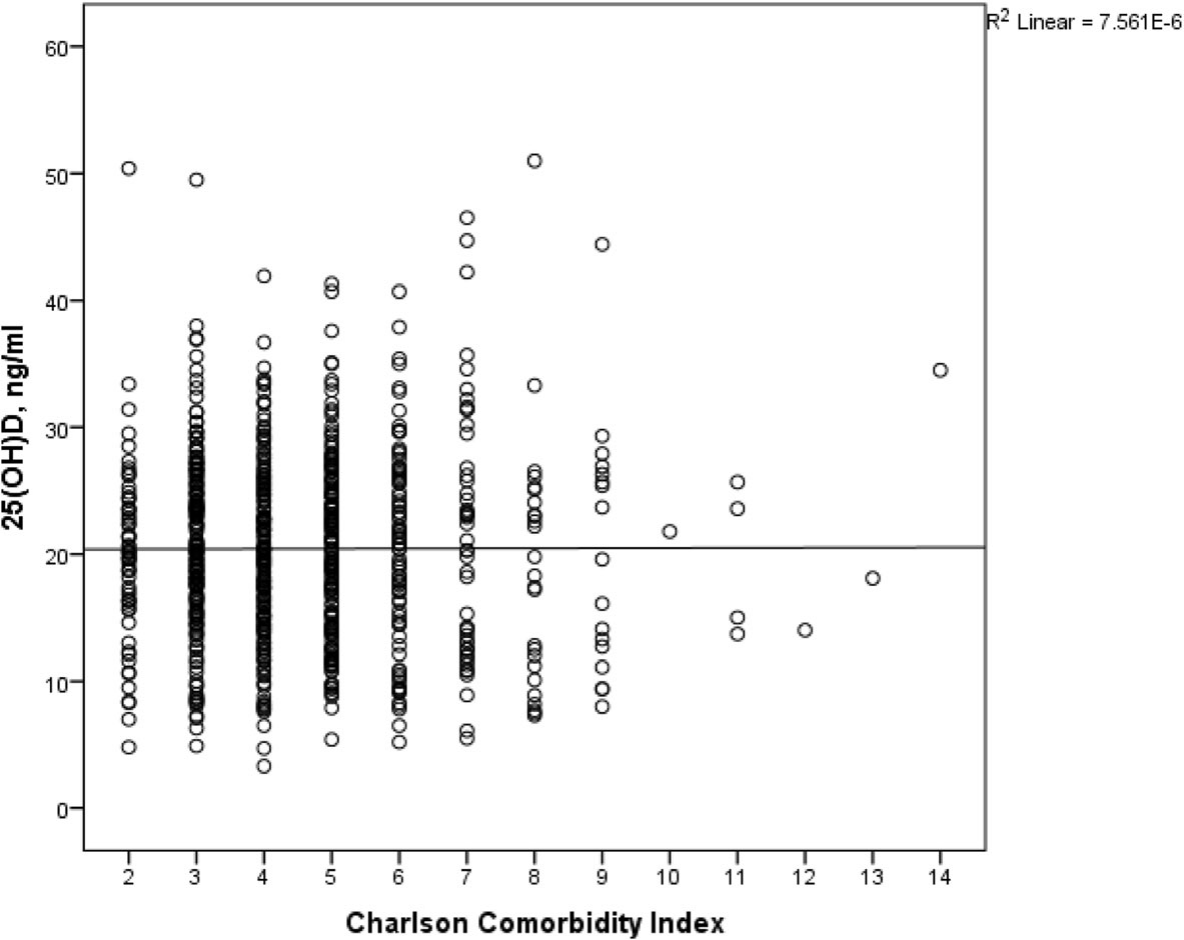
Scatter plot demonstrating the distribution of serum vitamin D level with respect to the Charlson Comorbidity Index

**Fig. 2:**
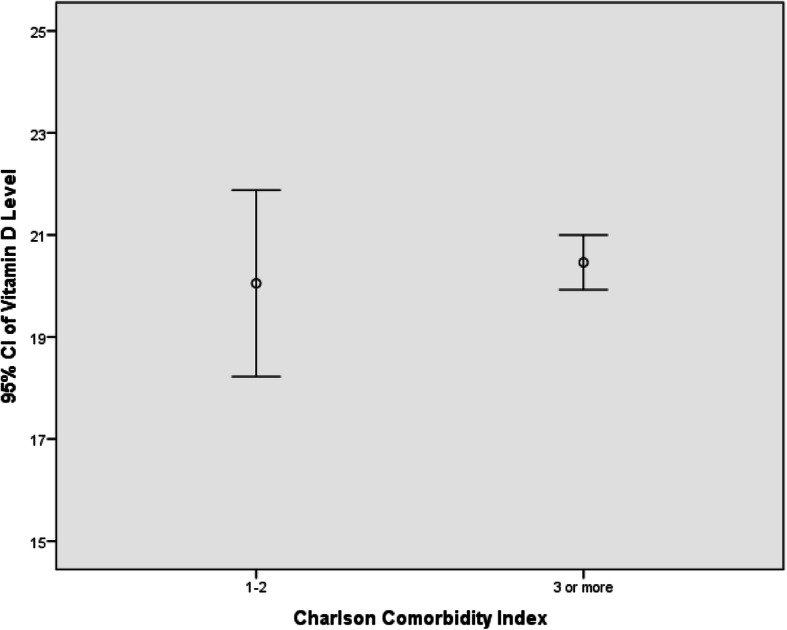
Error plot illustrating the distribution of mean vitamin D levels stratified by comorbidity burden

The mean age-adjusted CCI was 4.5 ± 1.8 years. The distribution of age-adjusted CCI with its corresponding mean vitamin D level is shown in Table 2. There was no correlation between the serum vitamin D level and the age-adjusted CCI (Pearson’s correlation coefficient = 0.003, *p* = 0.938) (Fig. 1). Approximately 7.8% of the patients with low comorbidity burden had a mean 25(OH)D level of 20.1 ± 7.2 ng/mL, and the remaining 92.2% of the patients with high comorbidity burden had a mean 25(OH)D level of 20.5 ± 7.4. After stratifying the CCI scores into these 2 groups, we did not find significant differences between the 2 subgroups for age-adjusted CCI and 25(OH)D levels (*p* = 0.497) (Fig. 2). Moreover, there was no association among age, gender and fracture types with the mean vitamin D level (*p* > 0.05). Of all the comorbidities in the geriatric hip fracture population, diabetes mellitus (DM) was the most prevalent comorbid condition (25.1%), followed by cerebrovascular accident (14.8%) and chronic renal disease (9.9%). Patients with DM with end-organ damage, chronic kidney disease, and congestive heart failure were found to have lower vitamin D levels (*p* < 0.05). In contrast, patients with dementia and cancer had significantly higher vitamin D levels (*p <* 0.05).

**Table 2:** The mean vitamin D level based on the Charlson Comorbidity Index and the individual comorbidities

Variables	***n*** = 796	%	25(OH)D, ng/ml (mean ± SD)	***p***-value
Age-adjusted CCI				
1–2	62	7.8	20.1 ± 7.2	
3–4	377	47.4	20.4 ± 6.9	0.971
5–6	257	32.3	20.5 ± 7.2	
≥ 7	100	12.6	20.5 ± 9.6	
DM				
No	596	74.9	20.4 ± 7.1	0.973
Yes	200	25.1	20.4 ± 8.1	
DM with end organ damage				
No	790	99.2	20.5 ± 7.4	0.009
Yes	6	0.8	12.6 ± 5.0	
Myocardial infarction				
No	771	96.9	20.5 ± 7.3	0.208
Yes	25	3.1	18.6 ± 9.3	
Congestive cardiac failure				
No	767	96.4	20.6 ± 7.3	0.015
Yes	29	3.6	17.1 ± 7.7	
Cerebrovascular accident				
No	678	85.2	20.3 ± 7.4	0.388
Yes	118	14.8	21.0 ± 7.3	
Hemiplegia				
No	768	96.5	20.4 ± 7.3	0.920
Yes	28	3.5	20.6 ± 8.6	
Dementia				
No	744	93.5	20.3 ± 7.3	0.010
Yes	52	6.5	23.0 ± 7.9	
COPD				
No	778	97.7	20.5 ± 7.4	0.197
Yes	18	2.3	18.2 ± 7.6	
Connective tissue disease				
No	100	100.0	20.4 ± 7.4	–
Yes	0	0.0	–	
PUD				
No	762	95.7	20.4 ± 7.4	0.710
Yes	34	4.3	20.9 ± 7.9	
Chronic kidney disease				
No	717	90.1	20.7 ± 7.2	0.016
Yes	79	9.9	18.1 ± 8.9	
Peripheral vascular disease				
No	783	98.4	20.4 ± 7.3	0.605
Yes	13	1.6	19.4 ± 9.4	
Cancer				
No	743	93.3	20.2 ± 7.2	0.001
Yes	53	6.7	23.7 ± 8.8	
Metastasis				
No	790	99.2	20.4 ± 7.4	0.806
Yes	6	0.8	21.2 ± 7.5	
Depression				
No	773	97.1	20.4 ± 7.3	0.751
Yes	23	2.9	21.1 ± 10.2	
Warfarin				
No	787	98.9	20.4 ± 7.4	0.837
Yes	9	1.1	20.9 ± 5.4	
Mild liver				
No	783	98.4	20.5 ± 7.4	0.188
Yes	13	1.6	17.8 ± 7.1	
Severe liver				
No	790	99.2	20.4 ± 7.3	0.717
Yes	6	0.8	22.8 ± 15.1	
AIDS				
No	794	99.7	20.4 ± 7.4	0.139
Yes	2	0.3	28.2 ± 12.5	

Abbreviations: *CCI* Charlson Comorbidity Index, *DM* diabetes mellitus, *COPD* chronic obstructive pulmonary disease, *PUD* peptic ulcer disease, *AIDS* acquired immunodeficiency syndrome

## Discussion

Our study findings suggested that virtually all elderly patients with hip fracture had low vitamin D levels. Only 8.3% of our patients had normal vitamin D levels. The high prevalence of 25(OH)D deficiency noted in our study was similar to other reports on elderly with hip fractures [4, 7, 28, 29]. The vitamin D level appeared unaffected by age, gender, and smoking status in our cohort, but it was significantly lower among Indian patients. This could have genetic or dietary implications and deserves further research.

Since the medical conditions associated with 25(OH)D deficiency are represented in the CCI and nearly all geriatric patients with osteoporotic hip fractures have multiple comorbidities, it would be logical to expect that patients with a higher comorbidity burden would be associated with a lower vitamin D level, i.e., a high CCI score correlated with a lower 25(OH)D level. Our study did not find any correlation between the vitamin D level and CCI scores in the elderly hip fracture population. To the best of our knowledge, this was the first study to analyse the relationship between CCI and vitamin D levels.

Vitamin D has gained much interest recently with the revelation of its role far beyond bone metabolism. Many studies have reported associations between suboptimal vitamin D levels and various comorbidities, including [1] cancers of the prostate, colon, pancreas, ovary, stomach, and breast [2]; cardiovascular diseases, including hypertension, myocardial infarction, and congestive heart failure [3]; diabetes mellitus [4]; infectious diseases, including HIV [5]; autoimmune diseases such as rheumatoid arthritis, Crohn’s disease, and multiple sclerosis [6]; stroke [7]; chronic obstructive pulmonary diseases; and [8] mental health issues [7, 9, 12–15, 17–21].

When the comorbidities were bundled and represented collectively as CCI, the association appears to be negated. The mean 25(OH)D level was 20.4 ± 7.4 ng/mL. We observed similar low vitamin D levels across all levels of CCI (Table 2). In other words, vitamin D deficiency is highly prevalent in the geriatric population with hip fracture, regulardless of the number of comorbidities. Hence, elderly patients with more comorbidities might not be associated with lower vitamin D level.

There are certain limitations that should be accounted for in this retrospective database study. Data on vitamin D levels were not available for 20% of the patients that were admitted for hip fracture. We did not have details that may have influenced the serum 25(OH)D status, such as medication details, physical activity and sun exposure. In addition, a single vitamin D level measurement may be an imperfect surrogate as a long-term indicator of the vitamin D levels. However, Singapore is extremely close to the equator and has uniform temperature and abundant sunshine throughout the year. This climatic condition minimizes the seasonal variation in circulating vitamin D level compared to that in countries distal from the equator, which has substantially varying vitamin D levels throughout the year, according to the season, in a nonlinear manner [3]. Although our study sample was large and represented the typical geriatric patient with hip fractures admitted to a hospital for treatment, our results cannot be extrapolated to other population groups [17]. The CCI might not be adequately sensitive to quantify the overall comorbidity burden in the geriatric hip fracture population. As the CCI is a composite index, it does not discriminate well among diseases and is not reflective of the severity of illness. Notably, the weight assigned to the individual comorbid conditions in CCI may not reflect their relative importance on the vitamin D level. One other possible explanation is that some of the associated conditions, such as inflammatory bowel diseases, are not part of the CCI score. Moreover, CCI includes some comorbidities that have no reported association with the vitamin D status, such as peptic ulcer disease, warfarin use, leukemia, lymphoma, metastasis, and several other solid cancers. The current literature seems to present more controversies than definitive conclusions with regard to the association of Vitamin D deficiency with various diseases and the role of vitamin D supplementation in preventive efforts. The level of 25(OH)D defined as vitamin D deficiency, remains controversial, and there is no consensus on the optimal level of 25(OH)D [30, 31]. Studies linking vitamin D status with the non-skeletal end-points arise from small observational case–control or cross-sectional experiments. The nature of the study design resulted in the identification of association and cannot prove a causal relationship [19]. The results from 34 intervention studies on vitamin D deficiency did not prove whether vitamin D supplementation is effective in reducing disease occurrence [32]. Recent large-scale studies continue to disprove the value of vitamin D supplementation in fracture prevention in community-dwelling older adults, cardiovascular protection, cancer prevention and diabetes [33–35]. The discrepancy between observational and interventional studies suggests that low vitamin D levels may only serve as a marker of ill health and are unlikely to be involved in the pathogenesis of diseases. Overall, the existing literature provides limited and conflicting support for any association between 25(OH)D status and non-skeletal health outcomes, and our current finding of a lack of association between CCI score and vitamin D levels would further underscore this issue.

In our study, DM with end-stage organ disease was significantly associated with lower vitamin D levels. There was no correlation between DM and vitamin D levels. Studies that examined the relationship between DM and vitamin D have reported inconsistent results. The association reported in some of these studies was limited to obese subjects, specific sex, and extremely low vitamin D levels [36, 37]. Consistent with some studies, we also observed lower vitamin D levels in patients with CCF [11]. Vitamin D is involved in multiple pathophysiological pathways relevant to heart failure. Data from large cohort studies have confirmed the association between vitamin D deficiency and the risk of CCF. However, other studies have not found any such association [38]. Vitamin D supplementation did not confer cardiovascular protection in a recent trial by Barbarawi et al. [39]. However, data from prospective clinical trials are lacking, and vitamin D supplementation does not decrease the risk of CCF and DM [12]. Dementia and cancer had significantly higher vitamin D levels in our study. This may be attributed to the routine vitamin D supplementation prescribed by the geriatricians and oncologists.

## Conclusions

Low vitamin D levels were highly prevalent in our fragility hip fracture cohort and were associated with ethnicity (Indian race). However, we found no relationship between the CCI scores and vitamin D levels in the geriatric hip population. Although there was some association between low vitamin D levels and specific disease conditions, the overall comorbid burden of patients with hip fractures did not seem to be a significant factor for low vitamin D levels.

## Data Availability

The datasets used and/or analysed during the current study are available from the corresponding author upon reasonable request.

## Abbreviations

CCI: Charlson Comorbidity Index
25(OH)D: 25-hydroxyvitamin D3
DM: Diabetes mellitus
COPD: Chronic obstructive pulmonary disease
PUD: Peptic ulcer disease
AIDS: Acquired immunodeficiency syndrome
